# Evaluating and Improving the Quality of Surgical Operative Notes at Elobeid Teaching Hospital: A Two-Phase Audit

**DOI:** 10.7759/cureus.80617

**Published:** 2025-03-15

**Authors:** Abubakr Muhammed, Abdarheem Mohamed Ibrahim Mohamed, Asim Faisal Awad Ibrahim, Mohamed Hassan Mohamed Suliman, Lina SeedAhmed, Nahid Siddig Mohmed Elhussein, Eman Esam Hassan Ali, Alshyma Alfatih Mohamed Abdallah, Safa Elhag Mustafa Awadelseed, Omer Abdellatif Ali Abdalla, Sumia Ahmed Suliman Ahmed, Eilaf Eltayeb Abdalla Abdelgadir, Tagwa Tag Elsir Mohamed Babiker, Mohamed Ahmed Hussein Ziada, Saddam Adam Alnour Mohammed, Ahmed Modawi Bakheet Jabraldar, Yageen Albasha Elshaieb Shabo, Sara Salah Abdallah Hassan, Abdullah Mohamed, Muntasir Bakhit

**Affiliations:** 1 Surgery, University of Gezira, Wad Madani, SDN; 2 General Surgery, Elobeid Teaching Hospital, Elobeid, SDN

**Keywords:** clinical audit, documentation quality, quality improvement, royal college of surgeons of england guidelines, surgical operative notes

## Abstract

Background: Surgical operative notes play a critical role in patient care, serving as legal documents and communication tools among healthcare professionals. However, inconsistencies in documentation can compromise patient safety and continuity of care. This study aimed to evaluate and improve the quality of surgical operative notes at Elobeid Teaching Hospital through structured interventions.

Methods: A two-phase audit was conducted at the hospital's Department of Surgery, comprising a retrospective phase followed by a prospective phase. In the retrospective phase, 50 operative notes were reviewed to establish a baseline for documentation completeness and accuracy. Following this, an intervention was implemented, which included the introduction of a standardized template based on the Royal College of Surgeons of England (RCSEng) guidelines and training sessions for surgical staff. In the prospective phase, 34 operative notes were analyzed to evaluate the impact of the intervention. The completeness and accuracy of documentation were assessed using predefined parameters in both phases.

Results: In the pre-intervention phase, significant deficiencies were noted in key documentation areas such as anticipated blood loss (0%), deep vein thrombosis (DVT) prophylaxis (2%), and postoperative care instructions (34%). Following the intervention, substantial improvements were observed, with compliance rates reaching 97-100% in several key parameters. The most notable improvements were seen in the documentation of anticipated blood loss (0-97%), prophylactic antibiotic use (20-100%), and DVT prophylaxis (2-82%).

Conclusion: Implementing a standardized documentation template and providing staff training significantly enhanced the quality of surgical operative notes. Continued auditing and reinforcement of best practices are recommended to sustain these improvements and further optimize patient care.

## Introduction

Surgical operative notes are fundamental to patient care, serving as critical legal documents and essential communication tools within multidisciplinary healthcare teams. The Royal College of Surgeons of England (RCSEng) emphasizes the importance of precise, thorough, and timely documentation of surgical procedures to ensure high-quality patient care and medicolegal protection [1]. These notes provide a comprehensive summary of the surgery performed, support postoperative management, and facilitate continuity of care among different healthcare providers. They are also crucial for clinical audits, quality improvement initiatives, and educational purposes, enhancing patient safety and professional growth [2].

Incomplete documentation in surgical operative notes can lead to significant legal and ethical challenges. Legally, inadequate records may result in disputes over the standard of care provided, potentially exposing healthcare providers to malpractice claims. Ethically, incomplete documentation compromises patient safety and continuity of care, as critical information may be missing, hindering effective postoperative management. Accurate and thorough operative notes are essential not only for medicolegal protection but also for upholding ethical standards in patient care, underscoring the importance of this study's objective [3]. The quality of these records affects medical documentation and the continuity of information essential for patient care. Besides their medical significance, the quality of operative notes carries economic and legal implications, making accurate documentation vital for both audits and enhancing patient care delivery [4]. As per RCSEng guidelines, operative notes should be completed promptly following the procedure to ensure no critical details are missed and to maintain a clear recollection of the surgery. These notes must be legibly and systematically written or dictated, including information on the procedure type, the surgeon's name, assistant details, surgical techniques, and any complications or deviations from the plan [1,4-6].

In numerous hospitals, especially those operating with limited resources, it is common for surgeons to rely on plain, unstructured paper for recording operative notes. This practice frequently leads to documentation that is both inconsistent and incomplete. The lack of standardized formats often results in the omission of crucial details, which can negatively impact patient care and undermine the legal reliability of medical records. Introducing structured templates, as advocated by the RCSEng, has the potential to greatly enhance the quality and uniformity of surgical documentation in such environments. By adopting these templates, hospitals can ensure that all necessary information is systematically recorded, thereby improving both clinical outcomes and the robustness of medical records [7].

Recent studies have highlighted the importance of standardized documentation in improving surgical outcomes. For instance, a study at Dongola Teaching Hospital in Sudan demonstrated that adherence to documentation standards improved from 50.3% to 71.9% after the introduction of a standardized template and staff training [8]. Similarly, a clinical audit at Doka Hospital showed a significant improvement in documentation quality, with compliance increasing from 50.5% to 82.5% following the implementation of a structured format and targeted training sessions [9]. These findings underscore the effectiveness of structured interventions in enhancing surgical documentation practices.

Given the critical role of operative notes in patient care and the documented benefits of standardized documentation, this study aims to evaluate and improve the quality of surgical operative notes at Elobeid Teaching Hospital. By adopting a structured approach similar to those used in previous studies, we seek to enhance the accuracy, completeness, and consistency of surgical documentation, ultimately improving patient outcomes and ensuring compliance with RCSEng guidelines.

## Materials and methods

Study design and setting

This clinical audit was conducted at the Department of Surgery of Elobeid Teaching Hospital in Sudan from August 1 to November 1, 2024. The study aimed to evaluate the quality of surgical operative notes and implement interventions to improve documentation practices.

Audit criteria and definitions

The audit evaluated the quality of surgical operative notes based on three key dimensions: accuracy, completeness, and consistency.

Accuracy was defined as the extent to which the operative notes reflected the actual surgical procedure, including correct details such as instruments used, intraoperative findings, and complications.

Completeness was assessed by verifying the inclusion of all essential elements required for comprehensive documentation, such as preoperative diagnosis, operative findings, procedures performed, anticipated blood loss, deep vein thrombosis (DVT) prophylaxis, prophylactic antibiotic administration, and postoperative care instructions.

Consistency referred to the uniformity of documentation across different notes and surgeons, ensuring that all critical information was recorded systematically.

Audit population and sample size

A comprehensive analysis was conducted on a total of 84 surgical notes. In the pre-intervention phase, 50 notes were examined retrospectively to establish a baseline for documentation quality. Following the implementation of the intervention, which included the introduction of a standardized template and staff training, 34 notes were reviewed prospectively in the post-intervention phase to assess improvements in documentation completeness and accuracy. To guarantee that the sample accurately reflected the diverse array of cases managed by the department, a straightforward random sampling method was employed. This approach ensured that the selected notes were representative of the full scope of surgical procedures documented, thereby providing a reliable basis for the audit. Importantly, the doctors were not aware that their documentation practices were being monitored during either phase of the audit, which helped minimize bias and ensured that the findings reflected routine clinical practice.

Data collection and analysis

The process of gathering data was meticulously executed by a team of trained medical professionals. These individuals carefully examined the surgical notes using a standardized checklist that was designed in accordance with the guidelines set forth by the RCSEng. This checklist was structured to evaluate 18 distinct criteria, with a primary focus on determining the thoroughness and precision of the documented information.

In the initial phase of the study, a retrospective analysis was conducted on 50 randomly chosen cases. Following this, a second phase was initiated, which involved a prospective review of 34 cases. This subsequent review took place after the introduction of an updated documentation template (proforma) and a series of training sessions aimed at educating staff on proper documentation practices. The objective of these measures was to enhance the quality and consistency of surgical records.

To assess the effectiveness of these interventions, the collected data was systematically analyzed using Microsoft Excel 2016 (Microsoft Corporation, Redmond, Washington, United States). This analysis aimed to identify and quantify any improvements in the quality of documentation between the two phases of the study, thereby providing insights into the impact of the revised proforma and training initiatives.

Pre-intervention Phase (First Cycle)

The pre-intervention phase began with a retrospective review of 50 randomly selected surgical operative notes from the Department of Surgery. 

Intervention Phase

To address the identified gaps in documentation, a comprehensive intervention was implemented, which included three primary components described below.

Creation of a standardized operation note format: The previously used unstructured format was replaced with a standardized template based on the RCSEng guidelines.

Dissemination of the new template: The standardized template was circulated throughout all surgical departments to achieve consistency in operation note documentation.

Instruction and training: Surgeons and surgical staff received training on the significance of comprehensive and standardized documentation. Training methods encompassed a Microsoft PowerPoint presentation (Microsoft Corporation, Redmond, Washington, United States) at departmental meetings, targeted group discussions, and one-on-one sessions to emphasize the correct use of the new template.

Post-intervention Phase (Second Cycle)

Following a one-month period of intervention, the subsequent phase of the audit commenced. Throughout this phase, 34 surgical operation records from Elobeid Teaching Hospital were examined and evaluated to measure the effects of the intervention. The objective was to appraise advancements in the documentation procedure and compliance with RCSEng standards.

Ethical considerations

Ethical clearance for this audit was obtained from the Institutional Review Board of Elobeid Teaching Hospital (approval number: Au0207-OTH). All personal and clinical information was anonymized before analysis to ensure privacy and confidentiality.

Statistical analysis

Data from both audit phases were analyzed using Microsoft Excel 2016. Compliance rates for each documentation criterion were calculated, and improvements between the first and second cycles were assessed. The results were presented in tables, highlighting the percentage improvement in each documentation parameter.

## Results

A total of 50 operative records were reviewed in the first cycle, followed by 34 records in the second cycle of this clinical audit. The quality of documentation significantly improved across multiple key domains following targeted interventions.

In the first cycle, compliance with key parameters varied, with notable deficiencies in the documentation of anticipated blood loss, which was recorded in 0 (0%), DVT prophylaxis in 1 (2%), prophylactic antibiotic administration in 10 (20%), and postoperative care instructions in 17 (34%). Additionally, the recording of problems/complications was documented in 9 (18%), and the rationale for extra procedures was included in 11 (22%).

Following the implementation of documentation improvement strategies, particularly the introduction of a standardized template based on the RCSEng guidelines, the second cycle demonstrated significant progress. Staff training sessions were also conducted, but the primary driver of improvement was the use of the standardized template, which enhanced the completeness and accuracy of surgical operative notes. Documentation rates for anticipated blood loss improved from 0 (0%) to 33 (97%), prophylactic antibiotic use increased from 10 (20%) to 34 (100%), and postoperative care instructions rose from 17 (34%) to 34 (100%). Similarly, substantial improvements were observed in the recording of DVT prophylaxis, which increased from 1 (2%) to 28 (82%), problems/complications, which rose from 9 (18%) to 25 (73%), and extra procedures and their rationale, which improved from 11 (22%) to 32 (94%).

Although some parameters, such as operative procedure details, showed a marginal increase from 44 (88%) to 33 (97%) and tissue removal documentation improved slightly from 39 (78%) to 28 (82%), the overall compliance trends indicate a marked enhancement in the completeness of operative records (Table 1). These findings highlight the effectiveness of the interventions while also identifying areas where further refinement may be needed to achieve consistent and comprehensive documentation.

**Table 1:** A summary of the frequency of documented parameters. DVT: deep vein thrombosis

Parameter	First cycle (n=50)	Second cycle (n=34)	Improvement
Date and time	22 (44%)	33 (97%)	53%
Type of operation	27 (54%)	32 (94%)	40%
Name of operator and assistant	40 (80%)	34 (100%)	20%
Name of anesthetist	28 (56%)	31 (91%)	35%
Operative procedure carried out	44 (88%)	33 (97%)	9%
Incision type	35 (70%)	34 (100%)	30%
Operative diagnosis	21 (42%)	34 (100%)	58%
Operative findings	34 (68%)	34 (100%)	32%
Problems/complications	9 (18%)	25 (73%)	55%
Extra procedures and reasons	11 (22%)	32 (94%)	72%
Details of tissue removed/added/altered	39 (78%)	28 (82%)	4%
Identification of prostheses/materials	13 (26%)	28 (82%)	56%
Details of closure technique	25 (50%)	31 (91%)	41%
Anticipated blood loss	0 (0%)	33 (97%)	97%
Prophylaxis antibiotics	10 (20%)	34 (100%)	80%
DVT prophylaxis	1 (2%)	28 (82%)	80%
Postoperative care instructions	17 (34%)	34 (100%)	66%
Surgeon's signature	20 (40%)	34 (100%)	60%

## Discussion

The findings of this audit highlight significant improvements in the quality of surgical operative notes at Elobeid Teaching Hospital following the implementation of structured interventions, including the introduction of a standardized template and targeted staff training. The results align with similar studies conducted in other healthcare settings, demonstrating the effectiveness of standardized documentation practices in enhancing the completeness and accuracy of operative notes [10].

The pre-intervention phase revealed significant documentation deficiencies, including the omission of anticipated blood loss (0%), DVT prophylaxis (2%), prophylactic antibiotic administration (20%), and postoperative care instructions (34%). These gaps have critical clinical implications: undocumented blood loss may lead to unaddressed intraoperative or postoperative bleeding; missing DVT prophylaxis records raise the risk of thromboembolic events; incomplete antibiotic documentation increases the likelihood of surgical site infections; and inadequate postoperative instructions may result in poor recovery or mismanaged complications. These omissions underscore the importance of accurate documentation in ensuring patient safety, adherence to clinical guidelines, and effective continuity of care, highlighting the study's relevance in addressing these gaps to improve both documentation quality and patient outcomes. These findings are consistent with studies conducted in other low-resource settings, where the absence of standardized templates often leads to inconsistent and incomplete records [8,11]. The introduction of a standardized template based on the RCSEng guidelines, coupled with staff training, resulted in significant improvements across all parameters. For instance, documentation rates for anticipated blood loss, prophylactic antibiotic use, and postoperative care instructions all reached 100% in the post-intervention phase. Similar improvements were observed in the recording of DVT prophylaxis (82%), problems/complications (73%), and extra procedures (94%) [9].

The success of these interventions mirrors findings from other studies. For example, a study at Dongola Teaching Hospital in Sudan demonstrated that adherence to documentation standards improved from 50.3% to 71.9% after the introduction of a standardized template and staff training [12]. Similarly, a clinical audit at Doka Hospital showed a significant improvement in documentation quality, with compliance increasing from 50.5% to 82.5% following the implementation of a structured format and targeted training sessions [9]. These studies underscore the effectiveness of structured interventions in enhancing surgical documentation practices, particularly in low-resource settings where the use of non-standardized methods is prevalent [10].

The improvements observed in this audit are also consistent with findings from studies conducted in other regions. For instance, a study at Port Sudan Teaching Hospital reported a significant increase in compliance with documentation standards from 51.9% in the first cycle to 82.1% in the second cycle following the implementation of an improved proforma and staff training [8]. Similarly, a study at Khyber Teaching Hospital in Pakistan revealed that the implementation of RCSEng guidelines led to a marked improvement in the documentation of perioperative findings, operative diagnosis, and team information, with compliance rates increasing from 70.8% to 95.7% [6]. These findings highlight the universal applicability of standardized documentation practices in improving the quality of surgical operative notes [11].

Despite the overall progress, certain areas, such as the documentation of the anesthetist's name and the surgeon's signature, showed only modest improvements. This is consistent with findings from other studies, which have identified persistent challenges in documenting certain parameters, particularly in settings where traditional documentation habits are deeply ingrained [11,13]. For example, a study in Wad Madani, Sudan, found that the name of the anesthetist was not documented in any of the operative notes reviewed, highlighting a systemic issue in documentation practices [11]. Addressing these challenges may require additional targeted interventions, such as the integration of electronic medical records (EMRs) and regular feedback sessions to reinforce the importance of comprehensive documentation [12].

The decline in the documentation of postoperative care instructions, from 100% in the first cycle to 90% in the second cycle, is a notable finding that warrants further attention. This decline may be attributed to the traditional practice of documenting postoperative instructions on separate sheets, as observed in other studies [8,4]. To address this issue, future interventions should emphasize the importance of including postoperative care instructions directly within the operative notes, ensuring that all relevant information is consolidated in a single document [9].

The findings of this audit also highlight the importance of regular audits and continuous quality improvement initiatives in maintaining high documentation standards. Studies have shown that ongoing audits and feedback mechanisms are crucial for sustaining improvements in documentation practices [13]. For example, a study at Arif Memorial Hospital in Lahore demonstrated that regular audits and the implementation of a redesigned proforma led to a 35.7% improvement in the documentation of operative notes [13]. Similarly, a study at Khyber Teaching Hospital found that the introduction of RCSEng guidelines and regular training sessions significantly improved the quality of operative notes, with compliance rates increasing from 70.8% to 95.7% [13].

The implementation of structured interventions, including standardized templates and staff training, has significantly improved the quality of surgical operative notes at Elobeid Teaching Hospital. However, continued efforts are needed to address persistent challenges, particularly in documenting certain parameters and ensuring the sustainability of improvements. Future interventions should focus on integrating electronic documentation systems, conducting regular audits, and providing ongoing training to healthcare professionals to ensure adherence to best practices in surgical documentation [12].

The audit has several notable limitations that could affect the scope and applicability of its findings. Firstly, it concentrates exclusively on one hospital, which raises concerns about the extent to which the results can be generalized or applied to other healthcare settings. Additionally, the study is primarily designed to assess the effects of introducing a new proforma, but it may fail to account for other simultaneous changes in clinical practices or shifts in patient outcomes that could influence the results. Furthermore, the audit places a strong emphasis on quantitative metrics, which might result in the neglect of important qualitative dimensions, such as the overall quality and depth of clinical documentation. These limitations suggest that the findings should be interpreted with caution and may not fully capture the broader context or nuances of the documentation process.

## Conclusions

The implementation of a standardized operative note template, combined with targeted staff training at Elobeid Teaching Hospital, led to a notable enhancement in the quality and completeness of surgical documentation. Critical elements such as anticipated blood loss, the use of prophylactic antibiotics, and postoperative care instructions demonstrated significant improvement after the intervention, reflecting a more structured and thorough approach to record-keeping. These results are consistent with existing evidence that highlights the benefits of standardized documentation systems in healthcare settings. However, while the intervention yielded positive outcomes, some gaps in documentation quality persisted, underscoring the need for sustained efforts to maintain and build upon these gains. To ensure long-term adherence to best practices, it is recommended that the hospital implement biannual audits to monitor documentation quality and identify areas for improvement. Additionally, ongoing education programs, such as quarterly workshops and refresher training sessions, should be established to reinforce best practices and address emerging challenges. Exploring the integration of EMRs could further streamline documentation processes and reduce errors. Such measures will not only help solidify the improvements achieved but also contribute to enhancing patient safety and the overall quality of care delivered. This study underscores the importance of structured documentation practices while emphasizing the need for continuous evaluation and refinement to achieve lasting impact.
